# Peptides: Molecular and Biotechnological Aspects

**DOI:** 10.3390/biom11010052

**Published:** 2021-01-01

**Authors:** Hamilton Cabral

**Affiliations:** Department of Pharmaceutical Sciences, School of Pharmaceutical Sciences of Ribeirão Preto, University of São Paulo, Ribeirão Preto, SP 14040-903, Brazil; hamilton@fcfrp.usp.br; Tel.: +55-16-3315-4970

Since the isolation and commercialization of insulin (a peptide composed of 51 amino acid residues) in the early 1920s, peptide drugs have reshaped the pharmaceutical industry [1], and their utilization continues to increase in medicine, biotechnology, and therapeutic research [2].

In 1950, Mellander was the first researcher to report bioactive peptides, suggesting that a set of casein-derived phosphopeptides increased vitamin D-dependent bone calcification in infants [3].

Bioactive peptides are amino acid sequences, ranging from 2 to 20 residues, that are hidden and inactive in proteins present in food [4]. These protein fragments present biological activity when released from the molecule of origin via hydrolysis by peptidases of the digestive system [5].

Bioactive peptides can be obtained through the action of peptidases secreted by microorganisms during the fermentation process in the presence of proteins; through hydrolysis using vegetal, animal, and microorganism peptidases; and through the gastrointestinal enzymatic processes [6]. Hydrolysis with peptidases is the most common method for the generation of bioactive peptides [6], whereby proteolytic enzymes, commonly called peptidases, catalyze the hydrolysis of peptide bonds. Each of these enzymes demonstrates a specificity towards each bioactive peptide [7].

Protein sources for peptide production are selected based on value-added proteins and amino acid sequences of pharmacological interest [8]. Thus, animal and vegetal enzymes are commonly used to obtain bioactive peptides [9], with most studies using milk, egg, meat, and fish proteins. The most common vegetal protein sources used in bioactive peptide production include soy, lentils, chickpeas, peas, beans, oats, and wheat [8]. Generally, peptides released by food protein hydrolysis present fewer side effects [10].

Bioactive peptides can present myriad biofunctionalities, depending on several characteristics, including amino acid sequences (extension, hydrophobicity, and load), and can also present multifunctionalities, thereby acting in different systems [4,11]. They modulate biological functions by interacting with specific target cell receptors, thereby triggering a beneficial physiological response in the body [12].

Several bioactive peptides obtained from various food sources, presenting multiple biological activities, have been reported in the literature [13]. Some of the activities have been reported in hypocholesterolemia [14] and dipeptidyl peptidase IV inhibition [15], along with antithrombotic, antioxidant [16], antihypertensive [17], antimicrobial [18], immunomodulatory [19], cytomodulatory [20], and antitumor [21] activities.

The use of omics techniques, including metabolomics, proteomics, and genomics, for analysis of toxins and other peptide sources can help identify bioactive peptides with unique structural characteristics generated by post-translational modifications [22]. Additionally, the development of bioinformatics and peptide databases is considered a precious strategy that can predict the action/function of these small molecules in organisms [23].

The safety of nano-encapsulated bioactive peptides should be carefully considered for toxicity related to increased functions and possible allergic reactions. To develop a safety evaluation strategy for bioactive proteins, other techniques can be used in addition to toxin and allergen examination, thereby increasing the efficiency of bioactive peptide evaluations in the future; analysis of the history of the protein source used, establishment of the structure–activity relationship of peptides using bioinformatics resources, and development of sensitive detection techniques for toxins and potential allergens that may coexist in peptide products [24] can be realized via advancements in techniques for such purposes.

The use of peptides in therapy has improved over time and will continue to advance drug development and treatment strategies, thereby setting new paradigms. Improvements in peptide screening and computational biology will continue to support the discovery of peptide drugs [22]. The potential of peptide drugs is enormous and, therefore, with recent advances in peptide science, the oncoming era may be called “The Age of Peptides” [25].
